# Species-specific identification of *Pseudomonas* based on 16S–23S rRNA gene internal transcribed spacer (ITS) and its combined application with next-generation sequencing

**DOI:** 10.1186/s12866-022-02607-w

**Published:** 2022-08-01

**Authors:** Shuqian Hu, Xiang Li, Xin Yin, Runmeng Li, Ruiyang Zhang, Junhao Zang, Yin Liu

**Affiliations:** The school of medicine, Nankai University, No.94 Weijin Road, Nankai District, Tianjin, 300071 China

**Keywords:** Bacterial identification, *Pseudomonas*, Internal transcribed spacer, ITS, Next-generation sequencing

## Abstract

**Background:**

*Pseudomonas* species are widely distributed in the human body, animals, plants, soil, fresh water, seawater, etc. *Pseudomonas aeruginosa* is one of the main pathogens involved in nosocomial infections. It can cause endocarditis, empyema, meningitis, septicaemia and even death. However, the *Pseudomonas* classification system is currently inadequate and not well established.

**Results:**

In this study, the whole genomes of 103 *Pseudomonas* strains belonging to 62 species available in GenBank were collected and the specificity of the 16S–23S ribosomal RNA internal transcribed spacer (ITS) sequence was analysed. Secondary structures of ITS transcripts determining where the diversity bases were located were predicted. The alignment results using BLAST indicated that the ITS sequence is specific for most species in the genus. The remaining species were identified by additional frequency analyses based on BLAST results. A double-blind experiment where 200 ITS sequences were randomly selected indicated that this method could identify *Pseudomonas* species with 100% sensitivity and specificity. In addition, we applied a universal primer to amplify the *Pseudomonas* ITS of DNA extracts from fish samples with next-generation sequencing. The ITS analysis results were utilized to species-specifically identify the proportion of *Pseudomonas* species in the samples.

**Conclusions:**

The present study developed a species-specific method identification and classification of *Pseudomonas* based on ITS sequences combined NGS. The method showed its potential application in other genera.

**Supplementary Information:**

The online version contains supplementary material available at 10.1186/s12866-022-02607-w.

## Background

The *Pseudomonas* was described in 1894 and is one of the most diverse and ubiquitous bacterial genera [1]. Detailed information about *Pseudomonas* is available at this link: https://lpsn.dsmz.de/genus/*pseudomonas*. *Pseudomonas* is a group of Gram-negative, and has both aerobic and anaerobic species [2], with a capsule and flagella. They are widely distributed in the human body, animals, plants, soil, fresh water, seawater, etc. [3–6]. The *Pseudomonas* genus is a metabolically versatile group of organisms, which are known to occupy numerous ecological niches. Their diversity influences the phylogenetic diversity and heterogeneity of these communities [7]. *Pseudomonas* consists of more than 200 effectively described species, most of which are closely related to clinical medicine. For example, *P. aeruginosa* is one of the most common pathogens causing infections in humans and warm-blooded animals, with the ability to migrate, evade host immune responses, damage host cells and be highly adaptable to a variety of environment [8]. It can lead to a wide range of disease manifestations, commonly chronic and difficult to eradicate. *P. aeruginosa* is considered as an opportunistic hospital-acquired Gram-negative pathogen, and is more likely to occur in immunocompromised populations. It can cause infection of airways, urinary tracts, skin and soft tissue associated with burns and surgery, eye and blood, especially for people with hypoimmunity [9]. It can also cause endocarditis, empyema, meningitis, septicaemia and even death. Infections of *P.aeruginosa* infections often manifest severe drug resistance, even causing death. Other strains of this genus are associated with fish pathogens that can cause haemorrhagic septicaemia and ulcer syndrome [10]. *P. fluorescens* can cause iatrogenic acute infection and has been reported in clinical samples from the oral cavity, stomach and lungs. Sepsis, septic shock and intravascular coagulation may occur after infection. As many antibiotics are not sensitive to *P. fluorescens*, the fatality rate is high [11]. Similarly, *P. stutzeri* has been shown to be associated with endocarditis [12]. In order to detect specific pathogens early and carry out clinical intervention as soon as possible, it is necessary to accurately and systematically identify *Pseudomonas* [13].

The traditional molecular detection of *Pseudomonas* usually focuses on specific genomic DNA sequences of one or several pathogens. These methods are not appropriate for systematic microecological analysis. Recently, next-generation sequencing (NGS) has been utilized as a powerful tool for pathogen detection and microecological research [14–17]. The target sequences for NGS bacterial analysis are variable regions in the 16S rRNA gene sequence [18]. Some new sequencing platforms can sequence approximate full-length 16S rRNA gene [19]. These methods can identify prokaryotes to the genus level, and have been widely used in the analysis of human and environmental microecology. However, a phylogenetic tree analysis of *Pseudomonas* demonstrated the low value of at least 30 species type strains to differentiate among species [20]. Nevertheless, genus identification usually cannot meet the microecological analysis needs. Although the physiological or biochemical characteristics of bacteria in the same genus are similar, their pathogenicity is quite different. For example, *P. aeruginosa* is an opportunistic pathogen causing a wide range of diseases in humans, whereas *P. stutzeri*, found in plants, rarely causes human diseases [21, 22].

Generally, a candidate target for exact phylogenetic analysis must depend on other alternative housekeeping genes of bacteria, such as *gyr*B, *rpo*D, and *rpo*B [23–25]. Meanwhile, the 16S–23S rRNA gene internal transcribed spacer (ITS) is a non-coding sequence in the *rrn* operon. It has also been utilized in bacterial phylogenetic analysis and identification [26]. As a non-coding sequence, the ITS is more specific than the 16S rRNA gene [27–30]. In our previous study, further analysis of BLAST results based on ITS sequences alignment were utilized to identified *Vibrio* and *Streptococcus* species-specifically [31–33].

The taxonomy of the *Pseudomonas* genus is controversial. In this study, we applied a method based on ITS for species-specific identification of *Pseudomonas.* Then, we actually utilized the method combined NGS to analyze a sample from fish. The results showed that *Pseudomonas* species indeed can be accurately identified at the species level. It may be applied to the detection of other prokaryotes.

## Results

### Primary and secondary structures

For this study, the complete genome sequences of 103 *Pseudomonas* strains from GenBank were collected and analyzed. These sequences belonged to 62 *Pseudomonas* species. The following is a summary of the ITS sequence characteristics of *Pseudomonas*. A total of 560 *rrn* operon sequences were collected and the number of ITSs in each strain was 3–8. In the *Pseudomonas* species selected, the number of operons was different (Supplemental Table 1). According to the tRNA gene (tDNA) contained in the ITS, all *rrn* operons can be divided into two types: (1) type N (ITS without tDNA), with a length of 310 ± 20 bp; and (2) type-IA (ITS contains tDNA^Ile^ and tDNA^Ala^), with a length of 500 ± 50 bp. Type-IA appears in all *Pseudomonas* species, whereas type N appears much less often, we selected the type-IA ITS as the research sequences. In addition, according to statistics, type-IA accounts for 91.1% of the 560 ITS sequences collected. The *Pseudomonas* species containing N-type ITS sequences were *P. putida*, *P. antarctica*, *P. entomophila*, *P. fulva*, *P. mandelii*, *P. plecoglossicida* and *P. psychrophila*, and their proportions of ITS types were different.

Type-IA ITS sequences were arranged and aligned using the GeneTool Lite 1.0 software and it showed a mosaic structure. These sequences were divided into five parts: (1) the upstream sequence of tDNA^Ile^ (US), with a length of 90 ± 30 bp; (2) tDNA^Ile^; (3) the linker sequence between tDNA^Ile^ and tDNA^Ala^ (LS), with a length of 20 ± 10 bp; (4) tDNA^Ala^; and (5) downstream of the tDNA^Ala^ sequence (DS), with a length of 240 ± 20 bp. All the US, LS, DS and the whole ITS sequence (WS) contain C regions and V regions. The N-type sequences of 13 strains were aligned, and the C regions and V regions could also be identified from the ITS sequence.

**Fig. 1:**
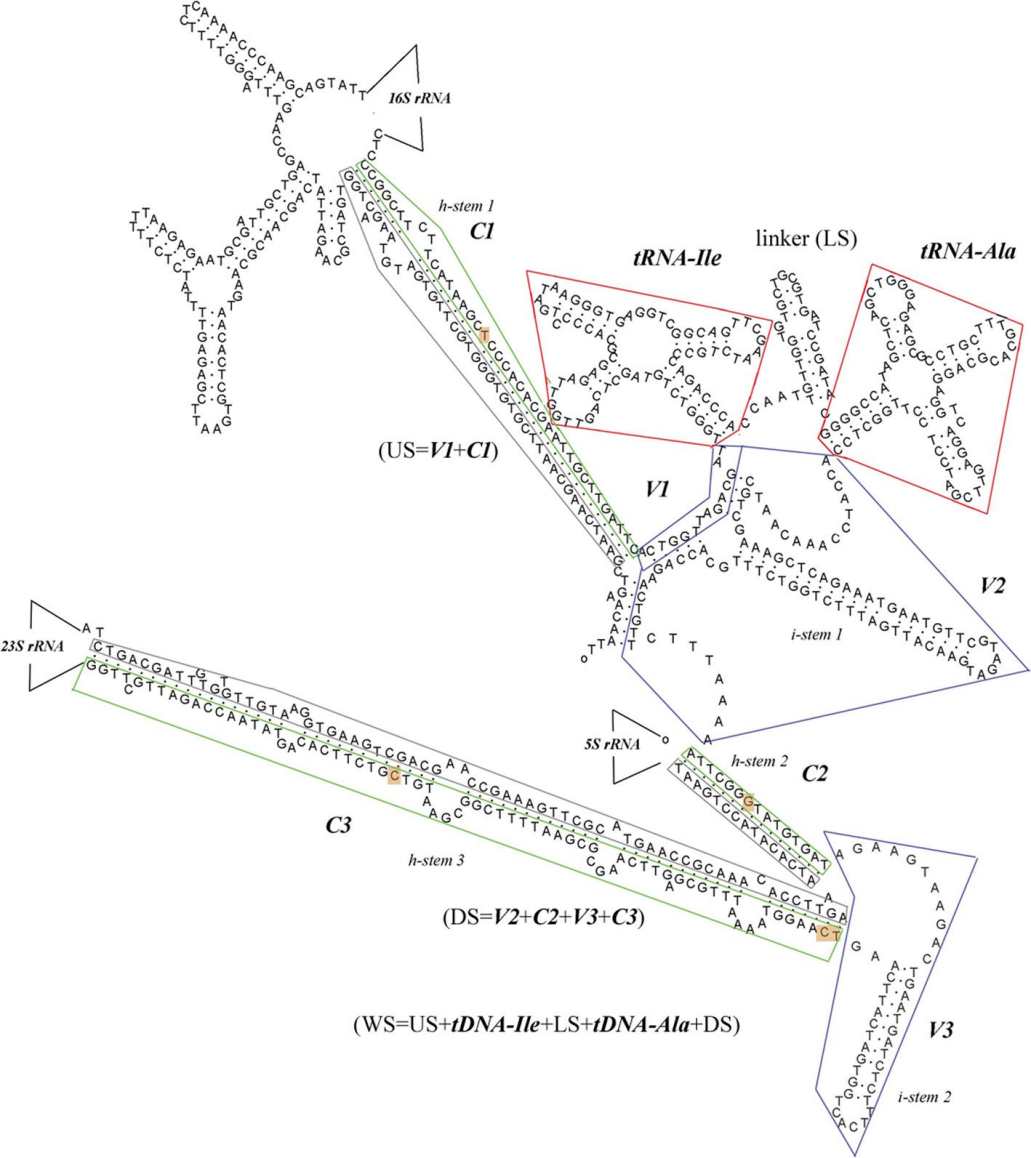
Secondary structures of *P. aeruginosa* ITS. Green frame, C1,C2,C3 block; gray frame, upstream of 16S rRNA gene and downstream of 23S rRNA gene; red frame, tRNA^Ile^ and tRNA^Ala^; blue frame, V block; orange frame, mutation region; I – stem, inner stem; H – stem, hybrid stem

After simulating the secondary structure of the type-IA *rrn* operon by RNA structure 4.2 software, the secondary structure of *Pseudomonas* species was found to share a common trunk. Taking *P. aeruginosa*, for example (Fig. 1), the secondary structure contains a variety of stem-loop structures. There are three hybridized stems respectively with the upstream of 16S rRNA gene or the downstream of 23S rRNA gene, constituting reverse complementary sequences called hybrid stems (h-stem), and two stems folded with the neighbouring sequences called inner items (i-stem). In addition, each ITS sequence contains three C regions (C1, C2, C3) and three V regions (V1, V2, V3). It corresponds to the mosaic structure obtained from the GeneTool Lite software. We determined that the diversity of the sequence was mostly in the inner stems. The ITS sequence participates in the folding of the 16S and 23S rRNA genes, indicating that the ITS is an important and indispensable structure.

### Species-specific analysis of ITS sequences

**Fig. 2:**
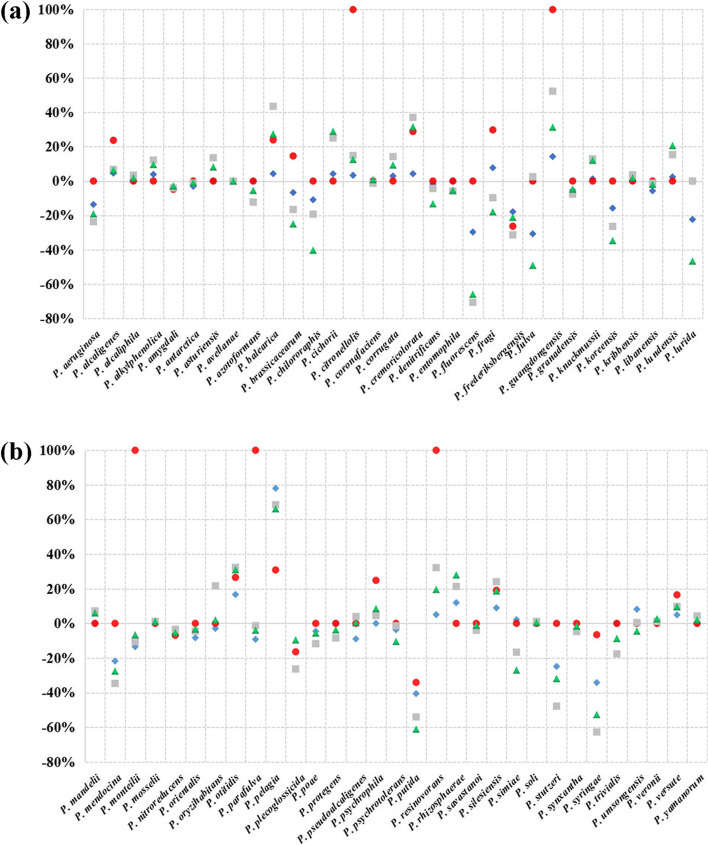
S-gap of *Pseudomonas* ITS. Vertical axis, S-gap value. Blue rhombic point, S-gap of US; red circular point, S-gap of LS; gray square point, S-gap of DS; green triangle point, S-gap of WS

The specificity of the four *Pseudomonas* substructures, which are US, LS, DS and WS, were evaluated by BLAST. The Gap values of 62 strains of *Pseudomonas* were obtained according to the RS values difference of the lowest target bacteria and the highest non-target bacteria in BLAST results (Fig. 2).

Although five red dots representing LS reached the Gap value of 100%, the short sequence caused no difference between target bacteria and non-target bacteria in BLAST results, often resulting in the Gap value of zero. This was shown in the figure of 41 strains, including *P. aeruginosa*, *P. denitrificans*, *P. entomophila*, *P. fluorescens*, *P. granadensis* and *P. knackmussii*. LS is not suitable as a species-specific DNA marker. In addition to the red dots, it can be clearly seen from the figure that the grey dots representing DS and the green dots representing WS show a higher Gap, whereas the blue dots representing US show a lower Gap because their sequence length is only about one-third of the DS.

By analyzing the results of Gap value, the US, DS and WS of 27 *Pseudomonas* species showed positive results, such as *P. alcaligenes*, *P. alcaliphila*, *P. asturiensis*, *P. balearica*, *P. corrugata*, *P. cremoricolorata*, etc. The ITS sequence used as a genetic marker in these 27 species was efficient. However, the Gap value of the other 35 species was negative number, and the performance of US, DS and WS was consistent*.*

Therefore, 35 strains were further analyzed and their frequency graphs were generated by calculating the RS values of target bacteria and non-target bacteria at each stage. According to the frequency analysis results of these 35 species, they can be divided into four types.

**Fig. 3:**
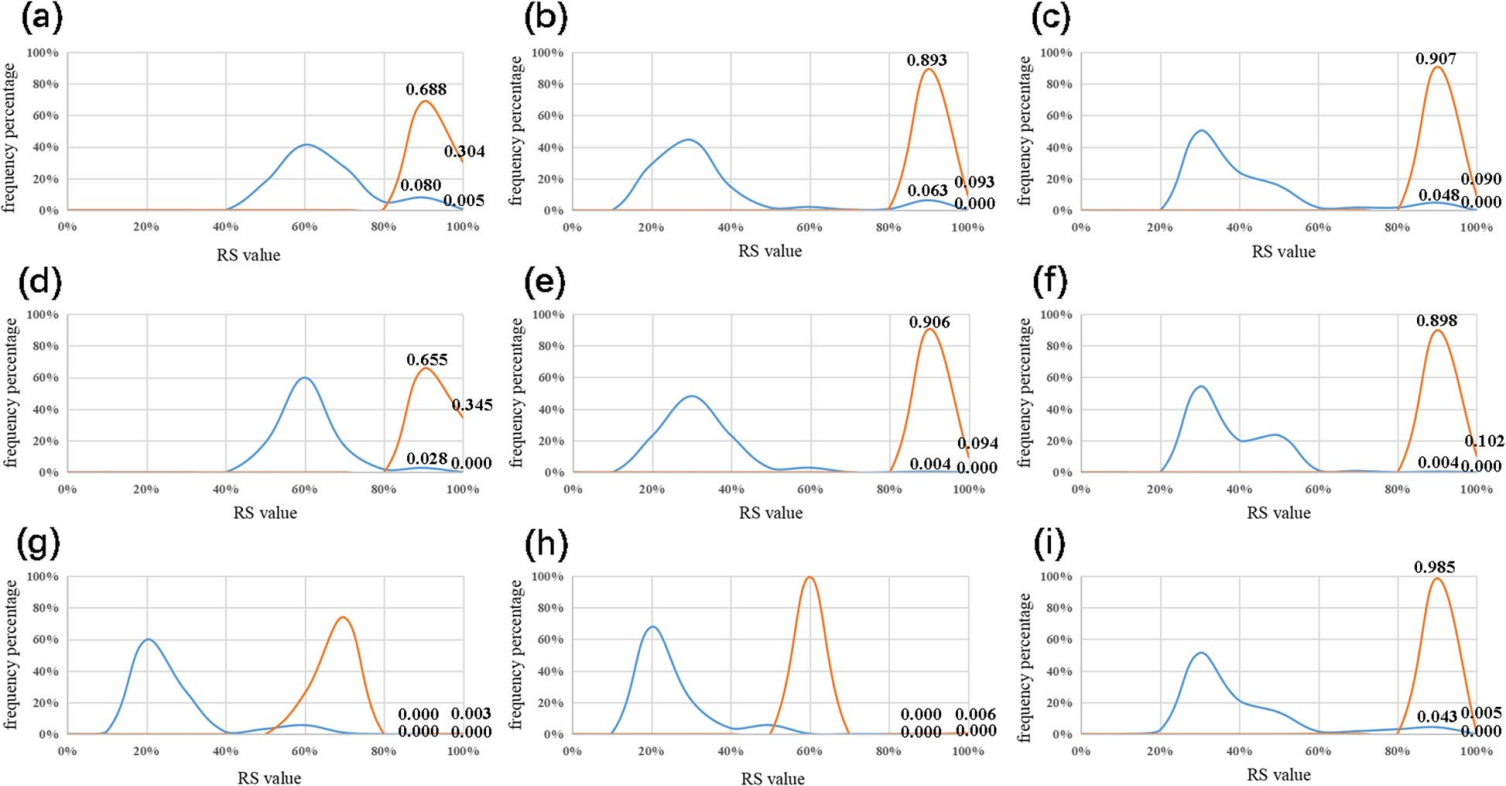
Frequency analysis of *P. aeruginosa.* Vertical axis, frequency value; horizontal axis, RS value. The red line, *P. aeruginosa* as the target species; the blue line, non-target species. **a** US of *P. aeruginosa* based on BLAST results*.* **b** DS of *P. aeruginosa.* **c** WS of *P. aeruginosa*. **d** US of *P. aeruginosa* based on genome-complete data. **e** DS of *P. aeruginosa*. **f** WS of *P. aeruginosa*. **g** WS of *P. aeruginosa* strain EPa3. **h** WS of *P. aeruginosa* strain G1. **i** WS of *P. aeruginosa* strain ATCC 10145 T

The first type. *P. aeruginosa* is the species with the largest amount of data in NCBI; the BLAST results are more complex as well. Its specificity was discussed in three aspects: (1) a frequency diagram was made based on BLAST results (Fig. 3a–c); (2) a frequency diagram was made with BLAST results containing only genome-complete data (Fig. 3d–f); and (3) the three *P. aeruginosa* sequences with low RS values from BLAST results were used as target bacteria to conduct BLAST again (Fig. 3g–i). The coverage of target bacteria and non-target bacteria was crossed, but the RS value of target bacteria aggregated on the horizontal axis was significantly higher than that of non-target bacteria. Therefore, frequency analysis can reveal the species-specificity of the ITS in *P. aeruginosa*. In addition, this conclusion can be obtained by frequency analysis of similar types of *P. putida, P. fluorescens, P. stutzeri,* etc.

**Fig. 4:**
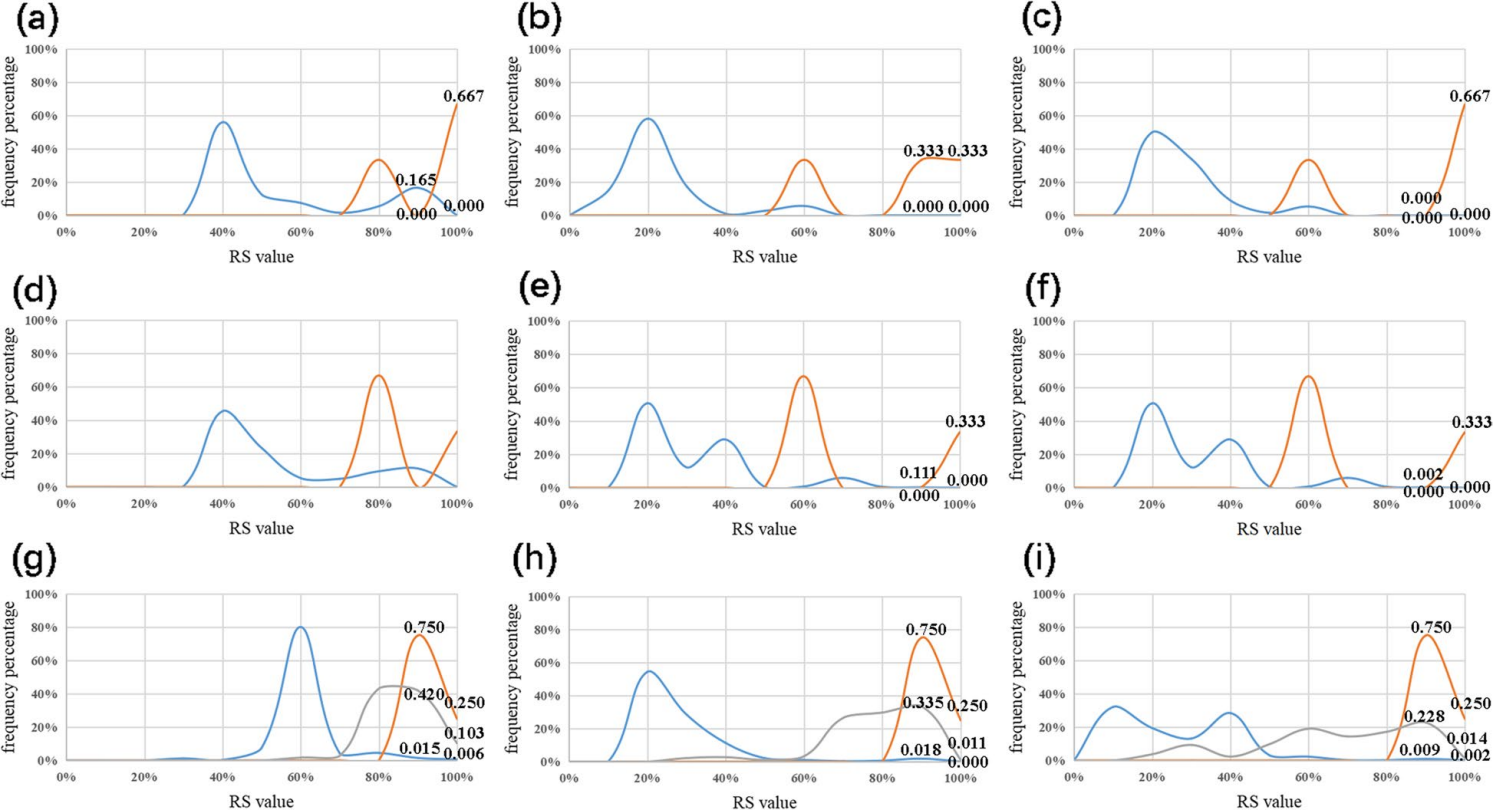
Frequency analysis of *P. parafulva* and *P. amygdali*. Vertical axis, frequency value; horizontal axis, RS value. **a**-**f** The red line, *P. parafulva* as the target species; the blue line, non-target species. **a** US of *P. parafulva*. **b** DS of *P. parafulva*. **c** WS of *P. parafulva*. **d** US of *P. parafulva* of lower RS values. **e** DS of *P. parafulva* of lower RS values. **f** WS of *P. parafulva* of lower RS values. **g**-**i** The red line, *P. amygdali* as the target species; the gray line: *P. syringae*; the blue line, other non-target species. **g** US of *P. amygdali*. **h** DS of *P. amygdali*. **i** WS of *P. amygdali*

The second type. The sequence of *P. parafulva* is few in NCBI database. In BLAST results, one of the ITS sequences showed a low RS value (0.62), making the RS of some non-target bacteria exceed it and showing a negative Gap value. In terms of this situation, the low RS sequence and the BLAST result were targeted to obtain six frequency diagrams (Fig. 4a–f). We still get good results from frequency analysis. This conclusion can be obtained from the analysis of the same type of *P. mordocina, P. monteilii and P. bassicacearum*.

The third type. From the BLAST results of *P. amygdali*, the GAP value of *P. amygdali* affected by *P. syringae* was shown. Instead of comparing two objects, *P. amygdali*, *P. syringae* and non-target bacteria were compared. The frequency diagram is shown in Fig. 4g–i. The RS value of *P. amygdali* represented by the red line aggregated on the horizontal axis was significantly higher than those of the other two groups, indicating the species-specificity of the ITS in *P. amygdali.*

**Fig. 5:**
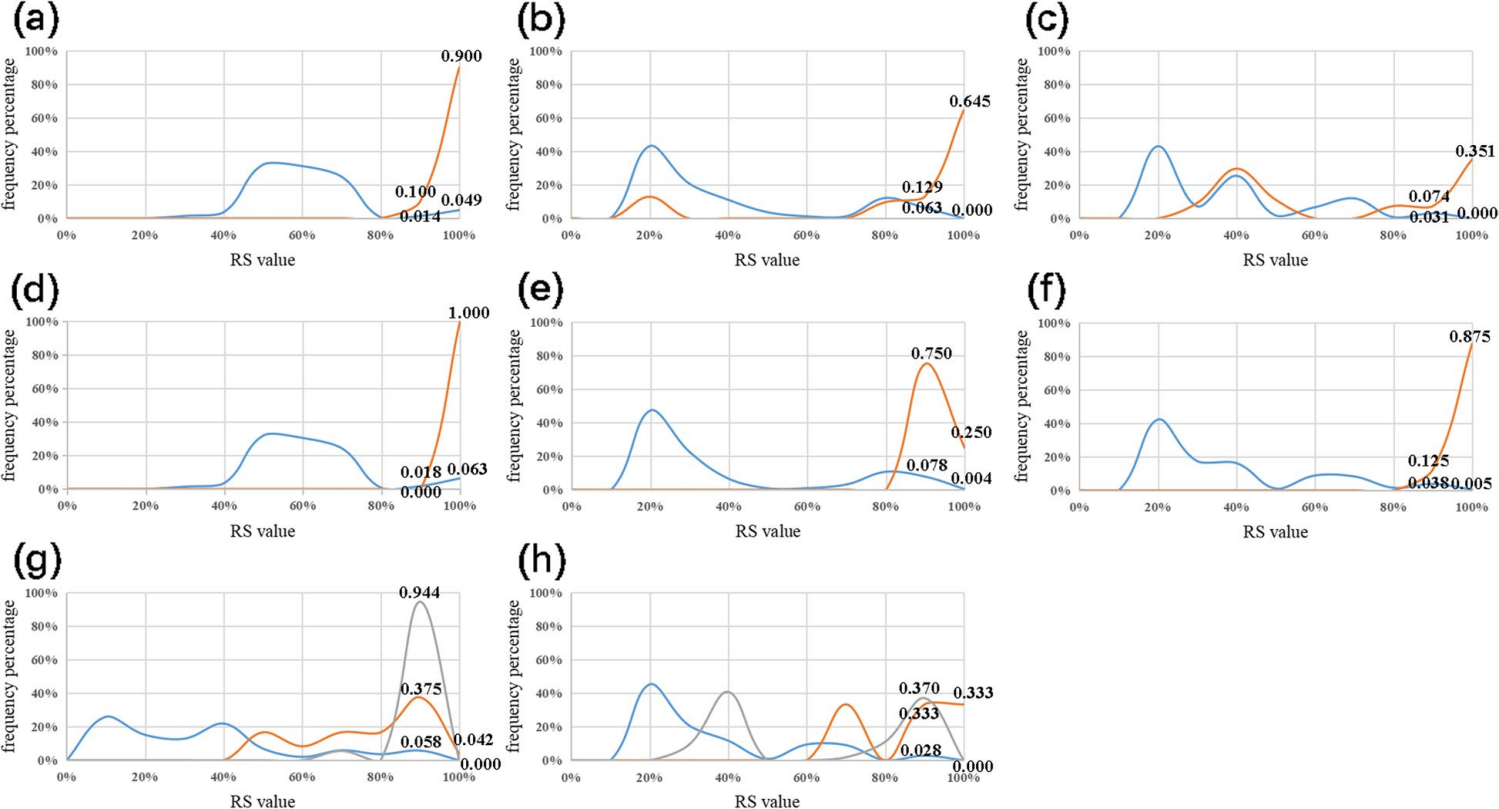
Frequency analysis of *P.syringae.* Vertical axis, frequency value; horizontal axis, RS value. a-f The red line, the target species; the blue line, non-target species. **a** US of *P. syringae pv. actinidiae*. **b** DS of *P. syringae pv. actinidiae*. **c** WS of *P. syringae pv. actinidiae*. **d** US of *P. syringae pv. tomato*. **e** DS of *P. syringae pv. tomato*. **f** WS of *P. syringae pv. tomato*. **g** WS of *P. syringae pv. syringae*. The red line, *P. syringae pv. syringae* as the target species; the gray line, *P. syringae pv. porri*; the blue line, other non-target species. **h** WS of *P. syringae pv. maculicola*. The Red line, *P. syringae pv. maculicola* as the target species; the gray line, *P. syringae pv. actinidiae*; the blue line, other non-target species

The fourth type. *P. syringae* can be pathogenic to a variety of organisms, which can be divided into *P. syringae pv. actinidiae*, *P. syringae pv. tomato*, *P. syringae pv. syringae* and other pathogenic bacteria. According to the frequency analysis, the species-specificity of the ITS in *P. syringae pv. actinidiae* and *P. syringae pv. tomato* was demonstrated (Fig. 5a–f). However, during the analysis of *P. syringae pv. syringae* and *P. syringae pv. maculicola*, their specificity could not be accurately obtained because of interference by other pathogenic bacteria (Fig. 5g, h). In conclusion, the ITS has high specificity with *P. syringae specie.*

Therefore, through frequency analysis with different ways of 35 species without GAP value, all species researched can be accurately distinguished by frequency chart. These results suggest that ITS and its subdomain can be used as DNA markers expressing species-specificity. These results this method is highly specific inter species, but not intra species.

### Identification of *Pseudomonas* by ITS

To verify this conclusion, 200 ITS sequences from the NCBI were selected, including ITS sequences of 160 *Pseudomonas* strains and ITS sequences of 40 non-*Pseudomonas* strains but belonging to *Pseudomonadales*, and then randomly scrambled for a double-blind experiment (Supplemental Table 2). In this experiment, neither the experimenter nor the analyst knew which strain the sequence belonged to. The results showed that 66 ITS sequences could be directly identified by Gap, and the remaining 134 ITS sequences were identified successfully by further frequency analysis, with a success rate of 100% (Table 1).

**Table 1:** Results of the double-blind experiment

	*Pseudomonas*	*Pseudomonadales* *Non-Pseudomonas*
Total	160	40
GAP	36	30
FRE	124	10
No distinction	0	0

^a^GAP, the amount of ITS sequences which could be identified by GAP

^b^FRE, the amount of ITS sequences which could be identified by further frequency analysis

### Identification of *Pseudomonas* sp. in samples

ITS sequences of the bacteria in samples were amplified, and species-level identification was performed. The ITS sequence analysis revealed the species and proportion of *Pseudomonas* in the samples. Abundance values of *Pseudomonas* were obtained from 12 samples. The three bacteria with the highest abundance in each sample and their proportions are listed in Table 2. *P. putida*, *P. monteilii*, *P. koreensis*, *P. aeruginosa* and *P. fluorescens* are widely distributed in water. Among them, *P. putida* is the most widely distributed, with the highest abundance.

**Table 2:** Results of next generation sequencing^a^

Sample number	*Pseudomonas* ITS / Total reads	Species1 (G/F)^b^		Species2 (G/F)		Species3 (G/F)	
1	9212 / 51,322	*P. putida* (F)	37%	*P. koreensis* (F)	17%	*P. fluorescens* (F)	6%
2	7321 / 46,231	*P. putida* (F)	51%	*P. monteilii* (G)	9%	*P. fluorescens* (F)	5%
3	8169 / 57,330	*P. putida* (F)	32%	*P. fluorescens* (F)	12%	*P. koreensis* (F)	9%
4	6949 / 37,655	*P. putida* (F)	21%	*P. aeruginosa* (F)	7%	*P. monteilii* (G)	5%
5	9333 / 50,137	*P. putida* (F)	52%	*P. fluorescens* (F)	11%	*P. monteilii* (G)	3%
6	6392 / 39,156	*P. putida* (F)	43%	*P. fluorescens* (F)	17%	*P. aeruginosa* (F)	11%
7	7396 / 51,203	*P. putida* (F)	35%	*P. aeruginosa* (F)	9%	*P. fluorescens* (F)	7%
8	8931 / 50,297	*P. putida* (F)	39%	*P. fluorescens* (F)	23%	*P. monteilii* (G)	6%
9	6569 / 40,365	*P. monteilii* (G)	17%	*P. putida* (F)	15%	*P. fluorescens* (F)	9%
10	9391 / 53,610	*P. putida* (F)	38%	*P. fluorescens* (F)	22%	*P. monteilii* (G)	14%
11	9474 / 55,238	*P. putida* (F)	51%	*P. koreensis* (F)	16%	*P. fluorescens* (F)	8%
12	6920 / 43,695	*P. putida* (F)	49%	*P. aeruginosa* (F)	11%	*P. fluorescens* (F)	6%

^a^Each sample is arranged in order of abundance from largest to smallest, and top 3 species are selected in every sample. The percentage after each specie is the abundance of the sample

^b^Gap or Frequency results can identify the sequence. The percentage is ratio of the ITS sequence of the species and the *Pseudomonas* ITS sequence

## Discussion

The NCBI has collected 1582 pieces of complete *Pseudomonas* genomes, which are divided into 98 species in Berger’s manual release 5.0 May 2004. Recently, analysis of the *Pseudomonas* genus suggested that rearrangement within the genus is needed. However, for more understandable, we still used the Berger’s manual system of *Pseudomonas* in this paper [20, 34, 35]. To ensure the randomness of species selection and to cover all species labelled and annotated as much as possible, 103 strains of *Pseudomonas* were selected. According to the primary structure comparison and secondary structure prediction, the variable region of the ITS sequence was preliminarily obtained in this genus to determine ITS diversity. Twenty-seven strains could be distinguished simply by Gap analysis and 35 strains could be distinguished by frequency analysis. Therefore, the conclusions drawn in this paper are more representative. During the study, this method could identify *Pseudomonas* to the species level, but also classify its pathovar, including *P. syringae pv. tomato*, *P. syringae pv. actinidiae*, *P. syringae pv. porri* and *P. coronafaciens pv. oryzae* (Fig. 5c, f). We used various software for analysis the sequences and evaluation the identification method developed in present study. The most important one of them is RNAstructure version 4.2, which illustrated the relationship between species-specific sequence in ITS and the secondary structure of ITS. We also arranged and compared the sequences by GeneTool version 1.0 and MEGA version 7.0. The GeneTool is faster, and MEGA is ready for a phylogenetic analysis. The GView Server is also employed to illustrated the ITS arrangement in the complete genome sequences. The output graphic of GView Server is more understandable and comprehensible than that of NCBI website. Due to the high similarity of 16S rRNA gene among *Pseudomonas* species [20], it is impossible to identify strains by 16S rRNA sequencing, which means it need to be verified by combining multiple genes. This is the innovation of this article. Although we have no method to verify our sequencing analysis results, we have made a full theoretical analysis in this article, and more samples can be combined to verify this method in future research. Currently, this method is still carried out manually. In addition, compared with previous research results on the distribution of *Pseudomonas* in water [36, 37], the NGS results obtained are simpler and more accurate for classification. This suggests that the ITS identification method combined with NGS has a wide scope of application. This study can provide a new genetic basis for updating the classification of *Pseudomonas*, and the combination of ITS and NGS is an effective tool for conducting microecological research. We will systematize and standardize the application of this technology to hospital laboratories, to accurately identify pathogens using molecular biology sequencing.

## Conclusion

The present study developed a species-specific method identification and classification of *Pseudomonas* based on ITS sequences combined NGS. The method showed its potential application in other genera.

## Methods

ITS sequence alignment and secondary structure prediction.

To analyze the ITS of *Pseudomonas* accurately, whole-genome sequences of 103 *Pseudomonas* strains were collected, belonging to 62 species, from the nucleotide database of the National Center for Biotechnology Information (NCBI) website (https://www.ncbi.nlm.nih.gov/nuccore). The accession numbers of these strains are listed in Supplemental Table 1. The complete genome sequences was analyzed and illustrated the ITS arrangement by the GView Server software (https://server.gview.ca/) [38]. All *rrn* operons in each genome were extracted and stored uniformly. The order of the *rrn* operon was arranged from 5′ to 3′ like the 16S rRNA gene, 23S rRNA gene and 5S rRNA gene; the 5′-end of 16S rRNA gene marked as (GAACTG); the 3′-end of 16S rRNA gene marked as (CCTTAA); the 5′-end and 3′-end of 23S rRNA gene marked as (GTTATA) and (ACAATT) respectively. Then, the ITS sequences were grouped based on their tDNA. The ITS sequences were analyzed using the GeneTool Lite 1.0 software. The consensus regions (C regions) and variable regions (V regions) were assessed by permutation. Then, the secondary structure of *rrn* transcript products was predicted using RNAstructure 4.2 software.

### ITS sequence specificity analysis

The whole ITS sequence (WS) was divided into three substructures by tDNA. These were the upstream sequence of tDNA^Ile^ (US), the sequence linking tDNA^Ile^ and tDNA^Ala^ (LS), and the downstream of tDNA^Ala^ sequence (DS). All subsequences were aligned with the WSs of each genome using BLAST with the default setting on the NCBI (https://blast.ncbi.nlm.nih.gov/Blast.cgi). Further analysis of BLAST results were performed [31, 39]. The Max Score was used as it covers the comprehensive results of the E value and other results. Briefly, the sequences in the BLAST result were divided into a target group (belonging to the same species as a query sequence) and a non-target group. The relative score (RS; defined as the ratio of the Max score of each sequence to the highest value of the Max score in every alignment) for each sequence. Then, the difference (Gap value) between the lowest RS value of target group and the highest RS value of non-target group was calculated. A positive Gap of any sequences(US, LS, DS and WS) would prove its species-specific. We analyze the results of the different sequences separately to determine which sequence is better at identifying the species.

**Fig. 6:**
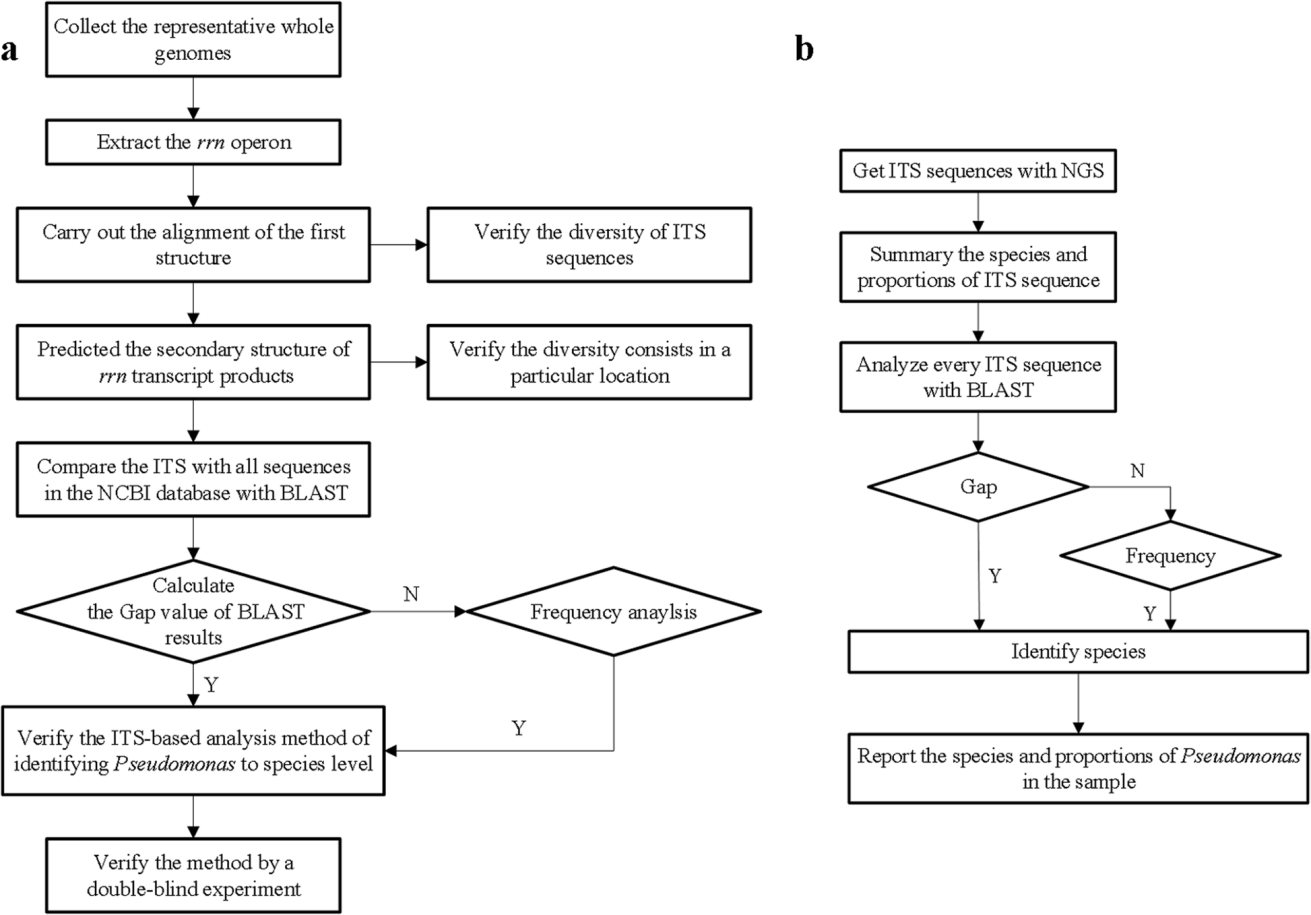
**a** Workflow for specific analysis of *Pseudomonas.* **b** Workflow for species identification of *Pseudomonas* by ITS sequence in the samples

For a negative Gap, further frequency analysis was performed. First, the RS values of target and non-target strains were grouped. Second, the RS values were divided from 0 to 100% into ten subgroups, with a spacing of 10%, and the RS = 100% sequence was separately divided into one subgroup, resulting in a total of 11 subgroups. Third, the number of sequences in each subgroup was calculated and the frequency of each subgroup sequence was determined. If the frequency of the target species is greater than that of non-target species in the RS = 100% subgroup, then we compared the frequency value in the next subgroup. If their frequencies are equal, the frequency values of the last subgroup can be compared. Then, these sequences are classified and a workflow can be developed for species-specific analysis of *Pseudomonas* (Fig. 6a).

### Verification ITS sequence specificity in *Pseudomonas*

To verify the Gap analysis and frequency analysis methods of identifying *Pseudomonas* to the species level, 160 sequences of *Pseudomonas* were selected as samples, labeled and scrambled; then, BLAST comparison, Gap analysis and frequency analysis were performed. The results were then compared with the original data to determine the specificity and sensitivity of this method. Specificity refers to the probability that the actual test result is undetected and correctly judged as undetected, denoted as SP; then, 1-SP = α is the probability of a false positive diagnosis. Sensitivity refers to the probability that the actual test result is correctly judged to be detected, denoted as Se; then, 1-Se = β is the probability of a false negative diagnosis. After that, 40 sequences of non-*Pseudomonas* genera but under *Pseudomonadales* were selected to arrange this method mentioned above, further demonstrating its effectiveness in higher classification elements.

### Application of the method for *Pseudomonas* in samples

A protocol for verifying the method was developed (Fig. 6b). To amplify the ITS sequences of *Pseudomonas*, a common primer set was designed (Pf, 5′-GAA GTC GTA ACA AGG TAG CCG TAG-3′ and Pr, 5′-AAC CGT CAG TCT CCG CTA CTT-3′). The primers can amplified the ITS sequences of *Pseudomonas* in the DNA samples. The species and proportion of ITS sequences in the DNA samples could be analysed based on the common primer combined with NGS using an Illumina NovaSeq 6000. For microecological analysis, we selected 12 DNA samples extracted from wild fish and identified *Pseudomonas* using the method mentioned in section 2.2.

## Supplementary Information

**Additional file 1: Table. S1.** Groups and the number of ITS in complete genome of *Pseudomonas.*

**Additional file 2: Table. S2.** Strains in the double-blind experiment.

**Additional file 3: Table. S3.** Identification process of Gap value for *P. balearica* by BLAST.

## Data Availability

The datasets generated and/or analysed during the current study are available in the NCBI repository (https://www.ncbi.nlm.nih.gov/nuccore).
