# Supplementary material for: Species-specific identification of Pseudomonas based on 16S–23S rRNA gene internal transcribed spacer (ITS) and its combined application with next-generation sequencing
Source: BMC Microbiol. 2022 Aug 1;22:188. doi: 10.1186/s12866-022-02607-w (PMC9341087; doi:10.1186/s12866-022-02607-w)
Supplement: Supplementary file 1 — Additional file 1: Table. S1. Groups and the number of ITS in complete genome of Pseudomonas. [file 12866_2022_2607_MOESM1_ESM.docx]

**Table. S1 Groups and the number of ITS in complete genome of *Pseudomonas.***

| Species | Strain | Number of ITS | | GeneBank |
| --- | --- | --- | --- | --- |
|  |  | IA^a^ | N^b^ |  |
| *P. aeruginosa* | Carb01 63 | 4 | 0 | CP011317 |
|  | F22031 | 4 | 0 | CP01206··6 |
|  | F30658 | 4 | 0 | CP008857 |
|  | F9676 | 4 | 0 | CP012066 |
|  | FRD1 | 4 | 0 | CP010555 |
|  | IOMTU 133 | 4 | 0 | AP017302 |
|  | M37351 | 4 | 0 | CP008863 |
|  | NCGM1900 | 4 | 0 | AP014622 |
|  | PA1RG | 4 | 0 | CP012679 |
|  | T63266 | 4 | 0 | CP008868 |
|  | VA-134 | 4 | 0 | CP013245 |
| *P. alcaligenes* | NEB 585 | 3 | 0 | CP014784.1 |
| *P. alcaliphila* | JAB1 | 4 | 0 | CP016162.1 |
| *P. alkylphenolica* | KL28 | 6 | 0 | CP009048.1 |
| *P. amygdali* | R15244 | 5 | 0 | CP026558.1 |
|  | ATCC 11528 | 5 | 0 | CP042804.1 |
|  | NM002 | 5 | 0 | CP020351.1 |
| *P. antarctica* | PAMC 27494 | 5 | 1 | CP015600.1 |
| *P. asturiensis* | CC1524 | 5 | 0 | CP047265.1 |
| *P. avellanae* | R2leaf | 5 | 0 | CP026562.1 |
| *P. azotoformans* | S4 | 6 | 0 | CP014546.1 |
|  | P45A | 6 | 0 | CP041236.1 |
| *P. balearica* | EC28 | 4 | 0 | CP045858.1 |
| *P. brassicacearum* | DF41 | 5 | 0 | CP007410.1 |
|  | LBUM300 | 5 | 0 | CP012680.1 |
|  | NFM421 | 5 | 0 | CP002585.1 |
| *P. chlororaphis* | PA23 | 5 | 0 | CP008696.1 |
|  | Lzh-T5 | 5 | 0 | CP025309.1 |
|  | UFB2 | 5 | 0 | CP011020.1 |
|  | JD37 | 5 | 0 | CP009290.1 |
|  | P2 | 5 | 0 | CP027719.1 |
|  | DSM 50083 | 5 | 0 | CP027712.1 |
|  | ZJU60 | 5 | 0 | CP027656.1 |
| *P. cichorii* | JBC1 | 6 | 0 | CP007039.1 |
| *P. citronellolis* | P3B5 | 5 | 0 | CP014158.1 |
| *P. coronafaciens* | X-1 | 5 | 0 | CP050260.1 |
|  | B19001 | 5 | 0 | CP046441.1 |
|  | 1_6 | 5 | 0 | CP046035.1 |
| *P. corrugata* | RM1-1-4 | 5 | 0 | CP014262.1 |
| *P. cremoricolorata* | ND07 | 6 | 0 | CP009455.1 |
| *P. denitrificans* | BG1 | 4 | 0 | CP043626.1 |
| *P. entomophila* | 1257 | 6 | 1 | CP034338.1 |
| *P. fluorescens* | UK4 | 6 | 0 | CP008896.1 |
| *P. fragi* | P121 | 5 | 0 | CP013861.1 |
|  | DBC | 2 | 6 | CP021986.1 |
| *P. frederiksbergensis* | AS1 | 4 | 3 | CP018319.1 |
|  | ERDD5:01 | 6 | 1 | CP017886.1 |
| *P. fulva* | FDAARGOS_167 | 4 | 3 | CP014025.1 |
|  | 12-X | 4 | 0 | CP002727.1 |
| *P. granadensis* | LMG 27940 | 6 | 0 | LT629778.1 |
| *P. guangdongensis* | CCTCC 2012022 | 5 | 0 | LT629780.1 |
| *P. knackmussii* | B13 | 4 | 0 | HG322950.1 |
| *P. koreensis* | D26 | 5 | 0 | CP014947.1 |
|  | BS3658 | 6 | 0 | LT629687.1 |
| *P. kribbensis* | 46-2 | 6 | 0 | CP029608.1 |
| *P. libanensis* | DMSP-1 | 6 | 0 | CP034425.1 |
| *P. lundensis* | AU1044 | 7 | 0 | CP017687.1 |
| *P. lurida* | MYb11 | 5 | 0 | CP023272.1 |
| *P. mandelii* | JR-1 | 5 | 1 | CP005960.1 |
| *P. mendocina* | MAE1-K | 4 | 0 | CP023641.1 |
|  | NEB698 | 4 | 0 | CP027657.1 |
| *P. monteilii* | USDA-ARS-USMARC-56711 | 6 | 0 | CP013997.1 |
|  | B5 | 3 | 4 | CP022562.1 |
| 1. *mosselii* | BS011 | 7 | 0 | CP023299.1 |
| *P. nitroreducens* | HBP1 | 5 | 0 | CP049140.1 |
| *P. orientalis* | F9 | 6 | 0 | CP018049.1 |
| *P. oryzihabitans* | USDA-ARS-USMARC-56511 | 5 | 0 | CP013987.1 |
| *P. otitidis* | MrB4 | 4 | 0 | AP022642.1 |
| *P. parafulva* | CRS01-1 | 7 | 0 | CP009747.1 |
|  | PRS09-11288 | 4 | 3 | CP019952.1 |
| *P. pelagia* | Kongs-67 | 3 | 0 | CP033116.1 |
| *P. plecoglossicida* | XSDHY-P | 3 | 4 | CP031146.1 |
| *P. poae* | CAP-2018 | 6 | 0 | CP034537.1 |
| *P. protegens* | H78 | 5 | 0 | CP013184.1 |
| *P. seudoalcaligenes* | CECT 5344 | 4 | 0 | HG916826.1 |
| *P. psychrophila* | KM02 | 5 | 3 | CP049044.1 |
| *P. psychrotolerans* | PRS08-11306 | 5 | 0 | CP018758.1 |
| *P. putida* | DLL-E4 | 1 | 4 | CP007620.1 |
|  | JBC17 | 6 | 0 | CP029693.1 |
|  | AA7 | 3 | 4 | CP018846.1 |
| *P. resinovorans* | NBRC 106553 | 5 | 0 | AP013068.1 |
| *P. rhizosphaerae* | DSM 16299 | 6 | 0 | CP009533.1 |
| *P.savastanoi* | NCPPB 3335 | 5 | 0 | CP008742.1 |
|  | 1448A | 5 | 0 | CP000058.1 |
| *P.silesiensis* | A3 | 5 | 2 | CP014870.1 |
| *P.simiae* | PCL1751 | 5 | 1 | CP010896.1 |
| *P.soli* | SJ10 | 7 | 0 | CP009365.1 |
| *P.stutzeri* | CGMCC 1.1803 | 4 | 0 | CP002881.1 |
|  | KGS-8 | 6 | 1 | CP018046.1 |
| *P.synxantha* | LBUM223 | 6 | 0 | CP011117.2 |
| *P.syringae* | inb918 | 6 | 0 | CP024646.1 |
|  | NZ-45 | 5 | 0 | CP017007.1 |
|  | LMG5095 | 5 | 0 | CP028490.1 |
|  | ATCC 10859 | 5 | 0 | CP013183.1 |
|  | ES4326 | 5 | 0 | CP047260.1 |
|  | PP1 | 5 | 0 | CP034078.1 |
|  | Pss9097 | 5 | 0 | CP026568.1 |
|  | B13-200 | 5 | 0 | CP019871.1 |
| *P.trivialis* | IHBB745 | 5 | 0 | CP011507.1 |
| *P.umsongensis* | CY-1 | 4 | 3 | CP051487.1 |
| *P.veronii* | R02 | 5 | 1 | CP018420.1 |
| *P.versuta* | L10.10 | 4 | 4 | CP012676.1 |
| *P.yamanorum* | LBUM636 | 5 | 0 | CP012400.2 |

a, ITS without tDNA b, ITS contains tDNA^Ile^ and tDNA^Ala^
