# Supplementary material for: Species-specific identification of Pseudomonas based on 16S–23S rRNA gene internal transcribed spacer (ITS) and its combined application with next-generation sequencing
Source: BMC Microbiol. 2022 Aug 1;22:188. doi: 10.1186/s12866-022-02607-w (PMC9341087; doi:10.1186/s12866-022-02607-w)
Supplement: Supplementary file 2 — Additional file 2: Table. S2. Strains in the double-blind experiment. [file 12866_2022_2607_MOESM2_ESM.doc]

**Table. S2 Strains in the double-blind experiment**

| Species | Accession |
| --- | --- |
| *Acinetobacter baumannii*^AB031^ | CP009256.1 |
| *Acinetobacter baylyi*^ADP1^ | CR543861.1 |
| *Acinetobacter bereziniae*^XH901^ | CP018259.1 |
| *Acinetobacter calcoaceticus*^CA16^ | CP020000.1 |
| *Acinetobacter chinensis*^WCHAc010005^ | CP032134.1 |
| *Acinetobacter cumulans*^WCHAc060092^ | CP035934.2 |
| *Acinetobacter defluvii*^WCHA30^ | CP029397.2 |
| *Acinetobacter dispersus*^NCCP 16014^ | CP041970.1 |
| *Acinetobacter equi*^114^ | CP012808.1 |
| *Acinetobacter gyllenbergii*^NCCP 16015^ | CP041971.1 |
| *Acinetobacter haemolyticus*^TJS01^ | CP018871.1 |
| *Acinetobacter indicus*^SGAir0564^ | CP024620.2 |
| *Acinetobacter johnsonii*^LXL_C1^ | CP031011.1 |
| *Acinetobacter junii*^65^ | CP019041.1 |
| *Acinetobacter lactucae*^OTEC-02^ | CP020015.1 |
| *Acinetobacter larvae*^BRTC-1^ | CP016895.1 |
| *Acinetobacter lwoffii*^ZS207^ | CP019143.2 |
| *Acinetobacter nosocomialis*^6411^ | CP010368.1 |
| *Acinetobacter oleivorans*^DR1^ | CP002080.1 |
| *Acinetobacter pittii*^AP_882^ | CP014477.1 |
| *Acinetobacter radioresistens*^DSSKY-A-001^ | CP027365.1 |
| *Acinetobacter schindleri*^ACE^ | CP015615.1 |
| *Acinetobacter soli*^GFJ2^ | CP016896.1 |
| *Acinetobacter tandoii*^SE63^ | CP041365.1 |
| *Acinetobacter towneri*^19110F47^ | CP046045.1 |
| *Acinetobacter wuhouensis*^WCHA60^ | CP031716.1 |
| *Azotobacter chroococcum*^B3^ | CP011835.1 |
| *Azotobacter salinestris*^KACC 13899^ | CP045302.1 |
| *Azotobacter vinelandii*^CA^ | CP005094.1 |
| *Moraxella bovis*^Epp63 (300)^ | CP030241.1 |
| *Moraxella bovoculi*^22581^ | CP011376.1 |
| *Moraxella catarrhalis*^25240^ | CP008804.1 |
| *Moraxella osloensis*^CCUG 350^ | CP014234.1 |
| *Moraxella ovis*^199/55^ | CP011158.1 |
| *Oblitimonas alkaliphila*^B4199^ | CP012358.1 |
| *Pseudomonas aeruginosa*^Carb01 63^ | CP011317.1 |
| *Pseudomonas aeruginosa*^F9676^ | CP012066.1 |
| *Pseudomonas aeruginosa*^CDN118^ | CP054591.1 |
| *Pseudomonas aeruginosa*^IOMTU 133^ | AP017302.1 |
| *Pseudomonas aeruginosa*^M37351^ | CP008863.1 |
| *Pseudomonas aeruginosa*^NCGM 1984^ | AP014646.1 |
| *Pseudomonas aeruginosa*^PA121617^ | CP016214.1 |
| *Pseudomonas aeruginosa*^T63266^ | CP008868.1 |
| *Pseudomonas aeruginosa*^VA-134^ | CP013245.1 |
| *Pseudomonas alcaligenes*^NEB 585^ | CP014784.1 |
| *Pseudomonas alcaliphila*^JAB1^ | CP016162.1 |
| *Pseudomonas alkylphenolica*^KL28^ | CP009048.1 |
| *Pseudomonas amygdali*^M301315^ | CP031225.1 |
| *Pseudomonas amygdali*^NM002^ | CP020351.1 |
| *Pseudomonas amygdali*^R15244^ | CP026558.1 |
| *Pseudomonas antarctica*^PAMC 27494^ | CP015600.1 |
| *Pseudomonas asturiensis*^CC1524^ | CP047265.1 |
| *Pseudomonas avellanae*^R2leaf^ | CP026562.1 |
| *Pseudomonas azotoformans*^S4^ | CP014546.1 |
| *Pseudomonas balearica*^EC28^ | CP045858.1 |
| *Pseudomonas brassicacearum*^3Re2-7^ | CP034725.1 |
| *Pseudomonas brassicacearum*^DF41^ | CP007410.1 |
| *Pseudomonas chlororaphis*^Lzh-T5^ | CP025309.1 |
| *Pseudomonas chlororaphis*^PA23^ | CP008696.1 |
| *Pseudomonas chlororaphis*^DSM 6698^ | CP027720.1 |
| *Pseudomonas chlororaphis*^DSM 50083^ | CP027712.1 |
| *Pseudomonas chlororaphis*^SLPH10^ | CP027710.1 |
| *Pseudomonas cichorii*^JBC1^ | CP007039.1 |
| *Pseudomonas citronellolis*^P3B5^ | CP014158.1 |
| *Pseudomonas coronafaciens*^1_6^ | CP046035.1 |
| *Pseudomonas coronafaciens*^X-1^ | CP050260.1 |
| *Pseudomonas corrugata*^RM1-1-4^ | CP014262.1 |
| *Pseudomonas cremoricolorata*^ND07^ | CP009455.1 |
| *Pseudomonas denitrificans*^BG1^ | CP043626.1 |
| *Pseudomonas entomophila*^1257^ | CP034338.1 |
| *Pseudomonas fluorescens*^MS82^ | CP028826.1 |
| *Pseudomonas fluorescens*^UK4^ | CP008896.1 |
| *Pseudomonas fragi*^P121^ | CP013861.1 |
| *Pseudomonas frederiksbergensis*^AS1^ | CP018319.1 |
| *Pseudomonas fulva*^FDAARGOS_167^ | CP014025.1 |
| *Pseudomonas furukawaii*^DNA^ | AP014862.1 |
| *Pseudomonas knackmussii*^B13^ | HG322950.1 |
| *Pseudomonas koreensis*^D26^ | CP014947.1 |
| *Pseudomonas kribbensis*^46-2^ | CP029608.1 |
| *Pseudomonas lundensis*^AU1044^ | CP017687.1 |
| *Pseudomonas lurida*^MYb11^ | CP023272.1 |
| *Pseudomonas mandelii*^JR-1^ | CP005960.1 |
| *Pseudomonas nitroreducens*^HBP1^ | CP049140.1 |
| *Pseudomonas orientalis*^F9^ | CP018049.1 |
| *Pseudomonas parafulva*^CRS01-1^ | CP009747.1 |
| *Pseudomonas putida*^AA7^ | CP018846.1 |
| *Pseudomonas putida*^H8234^ | CP005976.1 |
| *Pseudomonas putida*^IEC33019^ | CP016634.1 |
| *Pseudomonas putida*^PP112420^ | CP017073.1 |
| *Pseudomonas putida*^S12^ | CP009974.1 |
| *Pseudomonas putida*^W5^ | CP026115.2 |
| *Pseudomonas resinovorans*^NBRC 106553^ | AP013068.1 |
| *Pseudomonas rhizosphaerae*^DSM 16299^ | CP009533.1 |
| *Pseudomonas rhodesiae*^NL2019^ | CP054205.1 |
| *Pseudomonas savastanoi*^1448A^ | CP000058.1 |
| *Pseudomonas savastanoi*^NCPPB 3335^ | CP008742.1 |
| *Pseudomonas silesiensis*^A3^ | CP014870.1 |
| *Pseudomonas simiae*^PCL1751^ | CP010896.1 |
| *Pseudomonas soli*^SJ10^ | CP009365.1 |
| *Pseudomonas stutzeri*^A1501^ | CP000304.1 |
| *Pseudomonas stutzeri*^RCH2^ | CP003071.1 |
| *Pseudomonas stutzeri*^CGMCC 1.1803^ | CP002881.1 |
| *Pseudomonas stutzeri*^DW2-1^ | CP027543.1 |
| *Pseudomonas synxantha*^2-79^ | CP027755.1 |
| *Pseudomonas synxantha*^LBUM223^ | CP011117.2 |
| *Pseudomonas syringae*^ICMP 9853^ | CP018202.1 |
| *Pseudomonas syringae*^NZ-45^ | CP017007.1 |
| *Pseudomonas syringae*^LMG5095^ | CP028490.1 |
| *Pseudomonas syringae*^ATCC 10859^ | CP013183.1 |
| *Pseudomonas syringae*^ES4326^ | CP047260.1 |
| *Pseudomonas syringae*^PP1^ | CP034078.1 |
| *Pseudomonas syringae*^Pss9097^ | CP026568.1 |
| *Pseudomonas syringae*^B13-200^ | CP019871.1 |
| *Pseudomonas syringae*^USA011^ | CP045799.1 |
| *Pseudomonas trivialis*^IHBB745^ | CP011507.1 |
| *Pseudomonas umsongensis*^CY-1^ | CP051487.1 |
| *Pseudomonas veronii*^R02^ | CP018420.1 |
| *Pseudomonas versuta*^L10.10^ | CP012676.1 |
| *Pseudomonas yamanorum*^LBUM636^ | CP012400.2 |
| *Permianibacter aggregans*^HW001^ | CP037953.1 |
| *Pseudomonas aeruginosa*^CC1805^ | MK621020.1 |
| *Pseudomonas aeruginosa*^DPs-2^ | JN247777.1 |
| *Pseudomonas aeruginosa*^DPs-22^ | JN418884.1 |
| *Pseudomonas aeruginosa*^DPs-5^ | JN418876.1 |
| *Pseudomonas aeruginosa*^DVT401^ | CP050335.1 |
| *Pseudomonas aeruginosa*^SM9^ | KX641437.1 |
| *Pseudomonas aeruginosa*^TNAUAMH^ | DQ855959.1 |
| *Pseudomonas aeruginosa*^TPa2^ | KF202105.1 |
| *Pseudomonas alcaligenes*^BCRC 13909^ | EU014521.1 |
| *Pseudomonas avellanae*^R2leaf^ | CP026562.1 |
| *Pseudomonas azotoformans*^F77^ | CP019856.1 |
| *Pseudomonas balearica*^DSM 6083^ | CP007511.1 |
| *Pseudomonas brassicacearum*^L13-6-12^ | CP014693.1 |
| *Pseudomonas brassicacearum*^WMK10^ | MH269303.1 |
| *Pseudomonas chlororaphis*^B25^ | CP027753.1 |
| *Pseudomonas chlororaphis*^DSM 50083^ | DQ023306.1 |
| *Pseudomonas chlororaphis*^PCL1606^ | CP011110.1 |
| *Pseudomonas chlororaphis*^R47^ | CP019399.1 |
| *Pseudomonas chlororaphis*^464^ | CP027742.1 |
| *Pseudomonas chlororaphis*^M71^ | CP027744.1 |
| *Pseudomonas chlororaphis*^ChPhzTR39^ | CP027749.1 |
| *Pseudomonas chlororaphis*^DSM 6698^ | CP027720.1 |
| *Pseudomonas chlororaphis*^ChPhzS135^ | CP027738.1 |
| *Pseudomonas chlororaphis*^DTR133^ | CP027735.1 |
| *Pseudomonas entomophila*^2014^ | CP034337.1 |
| *Pseudomonas entomophila*^PS-PJH^ | FJ882924.1 |
| *Pseudomonas fluorescens*^B-909^ | AY582369.1 |
| *Pseudomonas fluorescens*^L111^ | CP015638.1 |
| *Pseudomonas fluorescens*^L321^ | CP015637.1 |
| *Pseudomonas fluorescens*^MPDS^ | CP054128.1 |
| *Pseudomonas fluorescens*^MS82^ | CP028826.1 |
| *Pseudomonas fluorescens*^DU3^ | CP032618.1 |
| *Pseudomonas fluorescens*^R-93-4^ | AY582364.1 |
| *Pseudomonas fluorescens*^R-915^ | AY582367.1 |
| *Pseudomonas fluorescens*^W-27-10^ | AY582363.1 |
| *Pseudomonas frederiksbergensis*^KNU-15^ | CP023466.1 |
| *Pseudomonas fulva*^SB1^ | CP023048.1 |
| *Pseudomonas granadensis*^PMK4^ | MH269305.1 |
| *Pseudomonas knackmussii*^B13^ | HG322950.1 |
| *Pseudomonas koreensis*^P19E3^ | CP027477.1 |
| *Pseudomonas lurida*^MYb11^ | CP023272.1 |
| *Pseudomonas mendocina*^S5.2^ | CP013124.1 |
| *Pseudomonas mendocina*^ATCC 25412^ | EU014571.1 |
| *Pseudomonas monteilii*^170918607^ | CP043395.1 |
| *Pseudomonas monteilii*^PtA1^ | CP024159.1 |
| *Pseudomonas orientalis*^8B^ | CP027723.1 |
| *Pseudomonas oryzihabitans*^Ep4^ | KM624633.1 |
| *Pseudomonas protegens*^CHA0^ | CP003190.1 |
| *Pseudomonas pseudoalcaligenes*^BCRC 11092^ | EU014553.1 |
| *Pseudomonas putida*^1A00316^ | CP014343.1 |
| *Pseudomonas putida*^SJTE-1^ | CP015876.1 |
| *Pseudomonas putida*^AA7^ | CP018846.1 |
| *Pseudomonas putida*^B1^ | CP022560.1 |
| *Pseudomonas putida*^BCRC 10459^ | EU014557.1 |
| *Pseudomonas putida*^BCRC 10460^ | EU014560.1 |
| *Pseudomonas putida*^JBC17^ | CP029693.1 |
| *Pseudomonas putida*^KH-1^ | EU717684.1 |
| *Pseudomonas putida*^QTH3^ | KU904402.1 |
| *Pseudomonas savastanoi*^ATCC 19304^ | AY342163.1 |
| *Pseudomonas stutzeri*^1W1-1A^ | CP027664.1 |
| *Pseudomonas stutzeri*^PM101005^ | CP046902.1 |
| *Pseudomonas stutzeri*^SLG510A3-8^ | CP011854.1 |
| *Pseudomonas stutzeri*^TPs1^ | KF202110.1 |
| *Pseudomonas synxantha*^R2-4-08W^ | CP027757.1 |
| *Pseudomonas syringae*^EFA 57^ | JF815536.1 |
| *Pseudomonas syringae*^ICMP 529^ | AY342170.1 |
| *Pseudomonas syringae*^B301D^ | CP005969.1 |
| *Pseudomonas syringae*^B-60-1^ | AY342175.1 |
| *Pseudomonas syringae*^ICMP 2452^ | AY342177.1 |
| *Pseudomonas syringae*^Ps25^ | CP034558.1 |
| *Pseudomonas syringae*^BD 348^ | AY644716.1 |
| *Pseudomonas syringae*^BP550^ | KX533932.1 |
| *Pseudomonas syringae*^LMG5185^ | AF209771.1 |
| *Pseudomonas syringae*^R-1/9^ | AY582358.1 |
| *Pseudomonas veronii*^Pvy^ | CP039631.3 |
| *Pseudomonas viridiflava*^Huanglv1^ | MH348157.1 |
| *Psychrobacter alimentarius*^PAMC 27889^ | CP014945.1 |
| *Psychrobacter arcticus*^273-4^ | CP000082.1 |
| *Psychrobacter cryohalolentis*^FDAARGOS_308^ | CP022043.2 |
| *Psychrobacter urativorans*^R10.10B^ | CP012678.1 |
